# Supplementary material for: National estimates of emergency department visits for pediatric severe sepsis in the United States
Source: PeerJ. 2013 May 21;1:e79. doi: 10.7717/peerj.79 (PMC3661074; doi:10.7717/peerj.79)
Supplement: Appendix S1 [file peerj-01-79-s002.docx]

# APPENDIX

Age-specific values for hypotension. ([Goldstein et al. 2005](#_ENREF_1))

| **Group** | **Age Range** | **Hypotension**  **mm Hg** |
| --- | --- | --- |
| Newborn | 0 days – 1 week | <59 |
| Neonate | 1 week – 1 month | <79 |
| Infant | 1 month – 1 year | <75 |
| Toddlers and Preschool | >1 year – 5 years | <74 |
| School age child | >5 years – 12 years | <83 |
| Adolescent and young adult | >12 years - <18 years | <90 |

Goldstein B, Giroir B, and Randolph A. 2005. International pediatric sepsis consensus conference: definitions for sepsis and organ dysfunction in pediatrics. *Pediatr Crit Care Med* 6:2-8.
